# Chloroplast DNA Copy Number Changes during Plant Development in Organelle DNA Polymerase Mutants

**DOI:** 10.3389/fpls.2016.00057

**Published:** 2016-02-04

**Authors:** Stewart A. Morley, Brent L. Nielsen

**Affiliations:** Department of Microbiology and Molecular Biology, Brigham Young UniversityProvo, UT, USA

**Keywords:** chloroplast DNA, DNA polymerase mutants, genome copy number, photosynthesis, quantitative PCR

## Abstract

Chloroplast genome copy number is very high in leaf tissue, with upwards of 10,000 or more copies of the chloroplast DNA (ctDNA) per leaf cell. This is often promoted as a major advantage for engineering the plastid genome, as it provides high gene copy number and thus is expected to result in high expression of foreign proteins from integrated genes. However, it is also known that ctDNA copy number and ctDNA integrity decrease as cells age. Quantitative PCR (qPCR) allows measurement of organelle DNA levels relative to a nuclear gene target. We have used this approach to determine changes in copy number of ctDNA relative to the nuclear genome at different ages of Arabidopsis plant growth and in organellar DNA polymerase mutants. The mutant plant lines have T-DNA insertions in genes encoding the two organelle localized DNA polymerases (PolIA and PolIB). Each of these mutant lines exhibits some delay in plant growth and development as compared to wild-type plants, with the PolIB plants having a more pronounced delay. Both mutant lines develop to maturity and produce viable seeds. Mutants for both proteins were observed to have a reduction in ctDNA and mtDNA copy number relative to wild type plants at all time points as measured by qPCR. Both DNA polymerase mutants had a fairly similar decrease in ctDNA copy number, while the PolIB mutant had a greater effect of reduction in mtDNA levels. However, despite similar decreases in genome copy number, RT-PCR analysis of PolIA mutants show that PolIB expression remains unchanged, suggesting that PolIA may not be essential to plant survival. Furthermore, genotypic analysis of plants from heterozygous parents display a strong pressure to maintain two functioning copies of PolIB. These results indicate that the two DNA polymerases are both important in ctDNA replication, and they are not fully redundant to each other, suggesting each has a specific function in plant organelles.

## Introduction

Through the process of endosymbiosis, ancient bacteria were engulfed by precursors of eukaryotic cells, and over time most of the genes required for organelle function from these ancestral bacteria have been moved into the nucleus. This raises the question, if most genes have migrated to the nucleus, why not all of them? How do chloroplasts benefit from maintaining their genomes? Most evidence suggests that the unique physiological environment of chloroplasts is required for proper regulation of chloroplast-specific genes. In a recent paper, John Allen (2015) proposes, supported by significant evidence from the literature, that redox regulation of gene expression is required within the membrane-bound compartment. A chloroplast sensor kinase may detect disruptions in the photosynthetic electron transport chain, which responds to changes in redox conditions to activate or repress chloroplast gene expression, allowing response and regulation of photosynthesis to changing environmental conditions (Allen, 2015). Light has been shown to affect the amount of chloroplast DNA (ctDNA) during plant development (Shaver et al., 2008). Evidence for regulation of chloroplast DNA (ctDNA) by the redox state of cells has been reported in *Chlamydomonas reinhardtii* (Kabeya and Miyagishima, 2013), and similarly for yeast mitochondrial DNA (mtDNA; Hori et al., 2009).

Despite the importance of these organelles, chloroplast and mitochondrial genomes possess relatively few of the genes required for their functions in photosynthesis and respiration. In *Arabidopsis thaliana* chloroplasts there are 87 protein-coding genes and 41 rRNA and tRNA genes (Sato et al., 1999). These numbers are very similar in chloroplast genomes from other higher plant species (Palmer, 1985). The organelle genomes require fully functional transcriptional and translational machinery for expression of the genes. However, plant organelles do not use nuclear DNA replication proteins. Instead, they utilize their own unique set of nuclear-encoded organellar localized DNA replication proteins to maintain their genomes. Many of these are dual-localized to chloroplasts and mitochondria (Christensen et al., 2005; Gualberto et al., 2013; Cupp and Nielsen, 2014; Moriyama and Sato, 2014).

In this paper we focus on chloroplast genome replication and maintenance. CtDNA in higher plants has been shown to replicate by a double-displacement loop mechanism from two specific replication origins (Kolodner and Tewari, 1975; Kunnimalaiyaan and Nielsen, 1997a,b) but may also replicate by a recombination-dependent (RDR) mechanism (Oldenburg and Bendich, 2004; Rowan et al., 2010; Nielsen et al., 2010). The use of two distinct replication mechanisms has been observed for many bacterial virus genomes (Kreuzer and Brister, 2010), where one mechanism is used during the initial stage of infection and another [RDR or rolling circle (RC) replication] for rapid replication of the phage genome for incorporation into new phage particles. The use of two or more mechanisms has been discussed as a possibility for ctDNA replication in plants (Nielsen et al., 2010). Replication via a double-displacement mechanism from specific origins may be involved in maintaining low levels of the chloroplast genome in mature or quiescent cells, while recombination-dependent replication may drive rapid replication to generate high copy numbers of the genome during early stages of plant development.

Tobacco (Ono et al., 2007) and Arabidopsis (Christensen et al., 2005; Parent et al., 2011) have been found to encode two closely related bacterial-like DNA polymerases, which have been designated PolIA and PolIB. Both are dual-localized to chloroplasts and mitochondria in these species (Christensen et al., 2005). PolIB has been shown to play a role in ctDNA repair (Mori et al., 2005; Parent et al., 2011) and mtDNA maintenance, photosynthesis, and respiration (Cupp and Nielsen, 2013). However, in rice (Kimura et al., 2002) and maize (Udy et al., 2012) a single chloroplast-localized DNA polymerase has been identified. By analysis of mutants the maize enzyme, encoded by the *w*2 gene, appears to be the only DNA polymerase that functions in chloroplasts and may also function in mitochondria (Udy et al., 2012). There is a paralog of this gene in maize, but the protein has not been detected in chloroplasts. Both maize proteins appear to be involved in mtDNA replication (Udy et al., 2012).

Although the identification and biochemical analysis of plant organelle-localized DNA polymerases has been progressing, limited research has been reported on the role and degree of redundancy of the two DNA polymerases that are found in Arabidopsis and some other species. We have examined the effects of mutations in the *A. thaliana* organellar DNA polymerases on ctDNA replication by quantitative PCR (qPCR) analysis of organelle DNA levels. We provide an analysis of the effects of T-DNA insertion mutations in either of the DNA polymerase genes on plant growth and development and chloroplast genome copy numbers.

## Materials and methods

### Planting and growing conditions

**Figure 1:**
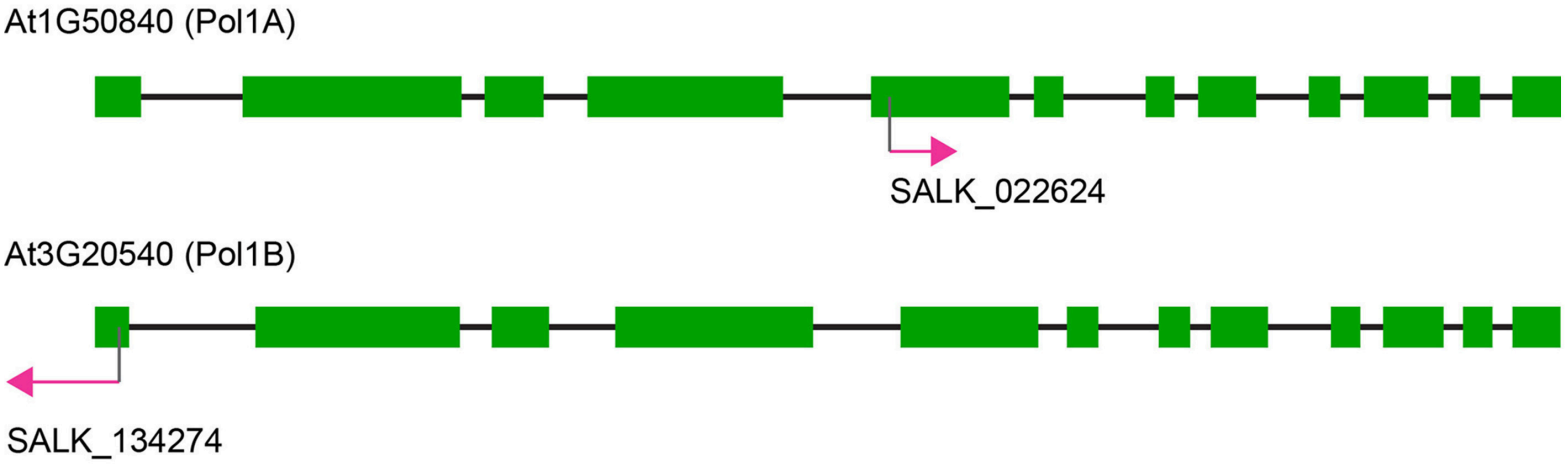
**Map of the DNA polymerase genes and T-DNA insertions**. Note the overall similarity between both genes for Pol1A and Pol1B. Both genes possess 12 exons although SALK_022624 inserts in the fifth exon of Pol1A whereas SALK_134274 inserts in the first exon of Pol1B.

We obtained the following T-DNA insertion lines from the Arabidopsis Biological Resource Center (Figure 1; ABRC; www.arabidopsis.org): Salk_022624 for PolIA (At1g50840); Salk_134274 (this is the same line designated polIb-1 in Cupp and Nielsen, 2013) for PolIB (At3g20540). Pots with the approximate dimensions 3 × 3 × 4 (width × length × height) inches were firmly packed with potting soil and placed in a tray. The soil was then saturated with nutrient water prepared with water-soluble fertilizer (Peter's Houseplant Food). Arabidopsis seeds were planted directly onto the surface of the soil and placed in a 4°C cold room in the dark for up to 3 days. Plants were then moved to a growth room maintained at 22°C with an average surface-light exposure of 80–100 μmol m^−2^ s^−1^. During the first 5 days of germination trays were covered with transparent plastic covers to maintain humidity and prevent drying, after which the covers were removed.

### Tissue harvesting and DNA extraction

Leaf tissue was harvested from plants at 7, 10, 14, and 21 dpi (days post-imbibition). Genomic DNA from these plants was then isolated following a cetyltrimethylammonium bromide (CTAB) method for isolating high quality DNA (Minas et al., 2011).

### Screening of T-DNA insertion lines

To determine if the T-DNA insertion was present, T-DNA specific primers were used in conjunction with native gene primers. Primers were designed so that native gene primers produced a PCR product about 1 kb in length, and that the T-DNA insertion primer paired with the native gene primer produced a PCR product ~500 b in length. Details of the primers used in zygosity screening are shown in Supplementary Table 1.

In order to obtain plants that were heterozygous for PolIA and PolIB genes, homozygous PolIA and PolIB plants were emasculated and then pollinated from either homozygous PolIA or PolIB flowers. This cross generated offspring that were heterozygous for both PolIA and PolIB, confirmed via PCR. Seeds from the first generation of heterozygous plants were collected to screen for all possible combinations of PolIA and PolIB using PCR as described above.

### Genome copy number analysis

Mitochondrial and chloroplast genome copy number was analyzed using an Applied Biosystems StepOne Plus qPCR machine and PowerUp SYBR green reagents. To analyze genome copy number, sequences unique to either ctDNA or mtDNA were identified. For ctDNA analysis, the targets psbK, petD, and ndhH were used. For mtDNA analysis, these targets included nad9, orf25, and cox1. The housekeeping gene AtRpoTp was used as a positive nuclear control and a reference for ΔΔCt calculations. A summary of these targets and their specific genes are listed in Supplementary Table 2. Technical and biological replicates were compiled and analyzed using the ΔΔCt method (Schmittgen and Livak, 2008; Cupp and Nielsen, 2013).

### Analysis of gene expression analysis in PolIA insertion line

mRNA was isolated from 7 dpi plants using PureLink Plant RNA Reagent (Life Technologies). RNase free DNaseI was added to remove residual DNA. Purity of mRNA was confirmed by running a small amount on a gel and checking for the absence of large DNA bands. cDNA for RT-PCR was generated from the purified mRNA using SuperScript III reverse transcriptase (Thermo Fisher). Primers for RT-PCR were designed to amplify a portion of the gene near the 3′ end of the mRNA. Primers for RT-PCR are described in Supplementary Table 3.

### Photosynthesis assays

Seeds from each mutant were germinated in plastic scintillation vials and grown under the same conditions as described above. At 14 dpi the vials were placed in a Licor 6400-22 Lighted Conifer Chamber Package connected to a Licor Li-6400XT analyzer. This system has the ability to measure photosynthetic rates and can automatically generate CO_2_ and light response curves. For this study, net photosynthetic rates of PolIA and PolIB mutants were calculated by measuring total leaf surface area. Total leaf area was calculated by scanning each plant and using ImageJ to trace and calculate surface area.

## Results

### Phenotype and expression analysis of organelle DNA polymerase mutants

**Figure 2:**
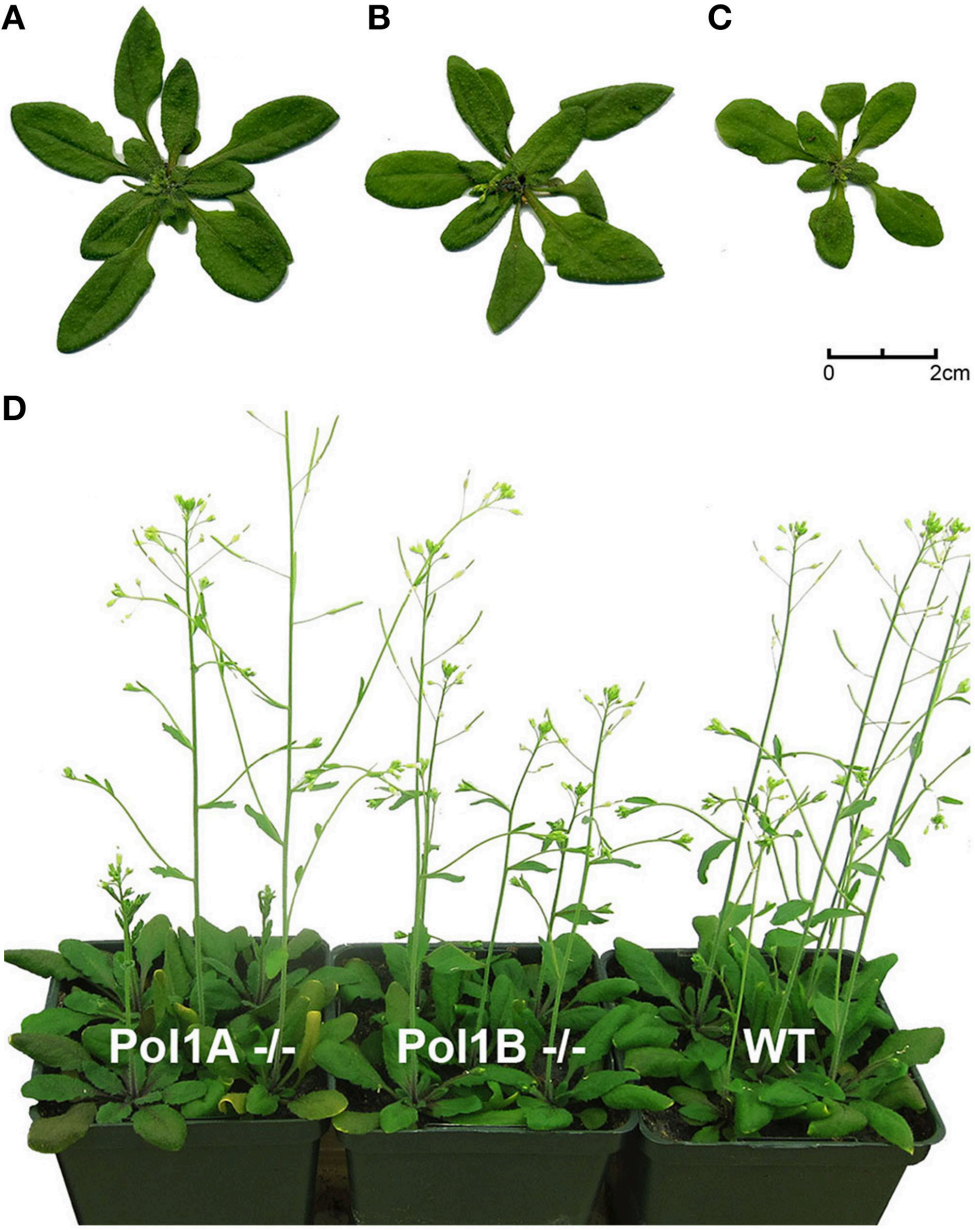
**Side by side comparison of 23 dpi WT (A)**, Pol1A^−/−^ **(B)**, and Pol1B^−/−^ **(C)** plants. Note the slightly delayed growth of Pol1B^−/−^ plants and the lack of a distinguishable phenotype between WT and Pol1A^−/−^ plants **(D)**.

The T-DNA insertion in PolIA is in the fifth exon of the gene, while the insertion in PolIB is in the first exon (Figure 1). The homozygous single mutant plants exhibited slight growth delays but both grow to maturity and produce seeds. Mutants in PolIB mutant plants exhibit a slower growth rate than the PolIA mutants. This pattern is consistent over time and reproducible (Figure 2; Supplementary Movie 1). This indicates that neither DNA polymerase is completely essential for development.

**Figure 3:**
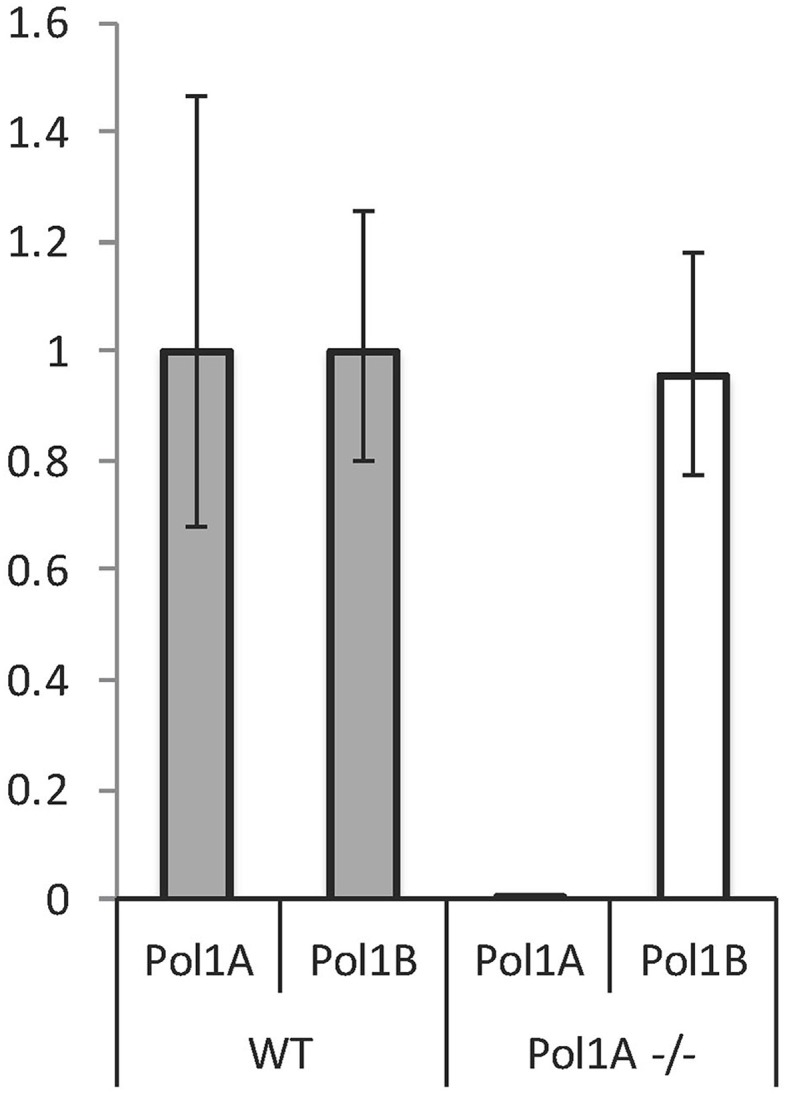
**RT-PCR of Pol1A and Pol1B expression in PolIA mutant plants**. Although previous work has suggested that mutation in Pol1B causes an increase in Pol1A expression, mutation of Pol1A does not affect expression of Pol1B. This experiment shows relative levels of each polymerase transcript normalized against Actin mRNA. Although mutation in Pol1A knocks down its expression, no significant change in Pol1B expression can be observed.

We previously showed that both DNA polymerases are expressed in most plant tissues during development, but there is a difference when comparing expression levels of the two genes. DNA PolIA is most highly expressed (relative to DNA PolIB) in rosette leaves, while DNA PolIB is expressed more abundantly in non-photosynthetic tissue (Cupp and Nielsen, 2013). We previously reported that in PolIB mutant plants, when expression of PolIB is knocked down a substantial increase (60–70%) in PolIA expression was observed by qRT-PCR analysis (Cupp and Nielsen, 2013). We were interested to determine if a similar compensatory effect occurs for the PolIA mutant. However, relative expression of PolIB in PolIA mutant plants was not significantly different from wild-type levels (Figure 3). This suggests an important role for DNA PolIA in chloroplasts and ctDNA maintenance, while PolIB may play a more significant role in mtDNA replication and maintenance.

**Figure 4:**
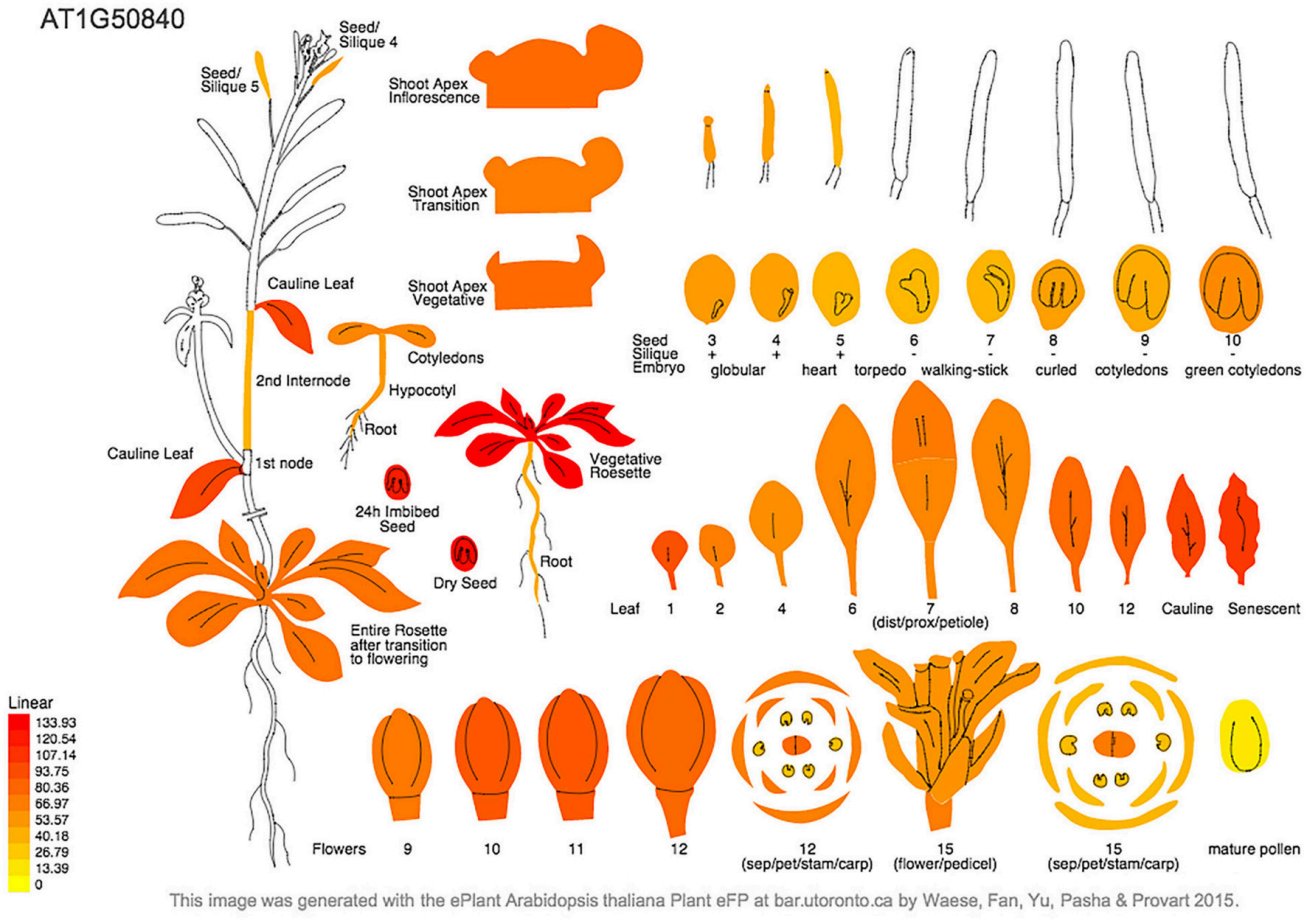
**Arabidopsis eFP browser report showing predicted PolIA gene expression in different plant tissues**. Expression of Pol1A is highest in rosette leaves, particularly at a young age, however, expression remains relatively high even in senescing leaves. Expression of Pol1A is lowest in seed embryos and pollen.

Our findings are consistent with expression of the Arabidopsis DNA PolIA gene compiled from microarray analysis in the Arabidopsis eFP browser (http://bar.utoronto.ca/~dev/eplant/). PolIA expression is highest in rosette leaves of wild-type plants, especially the youngest leaves, but is also high in imbibed seeds and developing flowers, and remains relatively high in cauline and older leaves. Expression of PolIA is low in embryos and siliques and in pollen (Figure 4), and is stimulated by drought and greatly repressed by osmotic stress (Nakabayashi et al., 2005; Schmid et al., 2005). Coexpression data (ATTED-II) indicates that the PolIA gene is coexpressed along with chloroplast-localized RecA, OSB2 (a single-stranded DNA binding protein, Gualberto et al., 2013) and some helicase genes. These proteins may all be involved in ctDNA replication, which would be compatible with the involvement of DNA recombination in chloroplast genome replication (RDR) and/or repair. There is very little information available for DNA PolIB in these databases.

Field-inversion gel electrophoresis (FIGE) and restriction pattern analysis of ctDNA from the mutants showed no discernable differences in the mutants compared to wild-type plants (data not shown). We used a PCR assay to detect any differences in rearrangement frequency in the mitochondrial genome, as has been observed for mutants affected in mtDNA recombination (Xu et al., 2011). However, the PolIA and PolIB mutants showed no differences in rearrangement frequency, indicating that there is no major disruption or change in the mechanism for DNA replication/recombination in the individual gene mutants for ctDNA or mtDNA (not shown).

### CtDNA and mtDNA copy number determination

**Figure 5:**
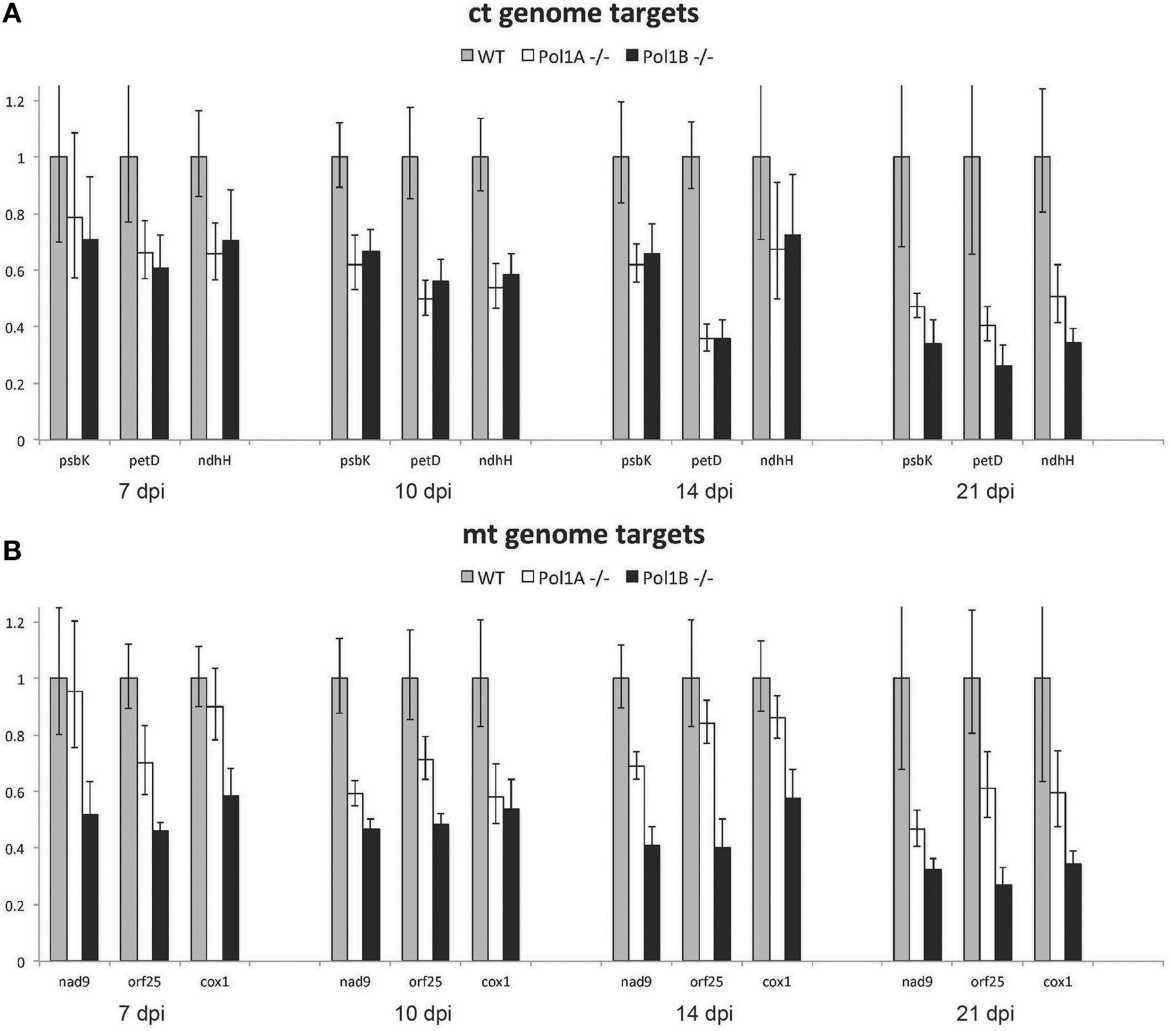
**Change in relative chloroplast and mitochondrial genome copy number**. Note that mutations in Pol1A and Pol1B affect chloroplast genome copy number equally **(A)** however mutation of Pol1B causes a more severe drop in mitochondrial genome copy number **(B)**. In both mutants, genome copy number gradually decreases but remains lower than wild type as the plants age.

qPCR analysis of ctDNA and mtDNA levels in each of the DNA polymerase mutant lines compared to wild-type showed that relative ctDNA levels and mtDNA levels, compared to the nuclear genome, are reduced in both PolIA and PolIB mutants, similar to what has been reported before for single time points (Parent et al., 2011; Cupp and Nielsen, 2013). To determine DNA levels at additional stages of growth, we analyzed samples at different time points. We examined DNA levels at 7, 10, 14, and 21 days of growth. At all time points there is a decrease in organelle DNA copy number in both mutants compared to wild-type plants of the same age for all 3 separate targets for each organelle genome at each age (Figure 5). Both PolIA and PolIB mutants showed a ~30% reduction in ctDNA at 7 days, a ~40% reduction at 10 and 14 days, and a 50% reduction at 21 days. At 21 days, there is a slightly greater reduction in the PolIB mutant (~60% decrease) compared to the PolIA mutant (~50% decrease). These results indicate that both DNA polymerases affect ctDNA copy number, in contrast with the finding in maize that a single DNA polymerase is responsible for ctDNA replication (Udy et al., 2012).

Similar but slightly different results were observed with the two mutant lines when mtDNA targets were analyzed. At 7 days the PolIA mutant showed only a slight drop in mtDNA copy number, while PolIB showed nearly a 40% drop (Figure 5), similar to what we previously reported (Cupp and Nielsen, 2013). At 10 and 14 days the PolIA mutant had a 20–40% drop in mtDNA copy number, while in PolIB the decrease was about 50%. At 21 days, the PolIA mutant had a 40% decrease in mtDNA, while the PoIB mutant showed a decrease of more than 60%. These results suggest that while both DNA polymerases contribute to mtDNA copy numbers, PolIB appears to play a greater role in maintenance of the mitochondrial genome. While qPCR analysis does not directly address quality of the DNA, it does show trends over time for the mutants compared to wild-type plants, indicating changes in organelle DNA levels during development in the mutants compared to wild-type plants.

### Analysis of photosynthesis in DNA polymerase mutants

**Figure 6:**
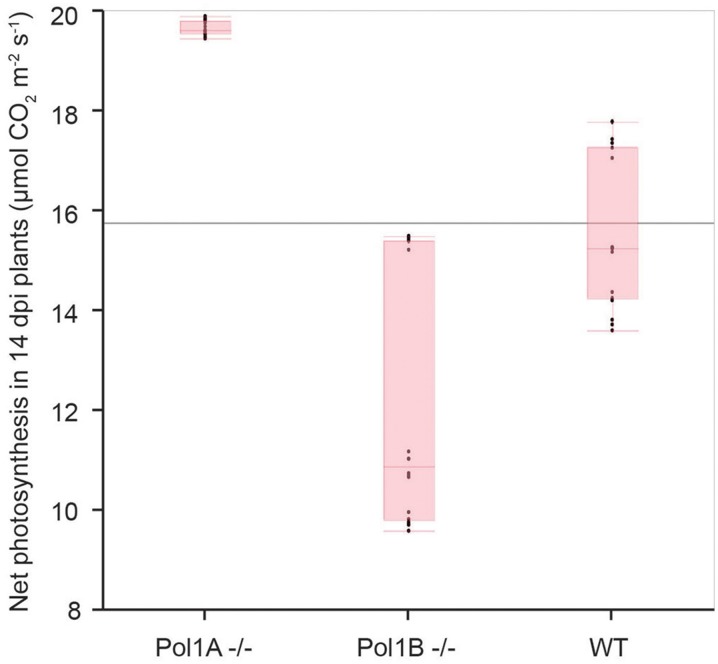
**Net photosynthetic rates in mutant plants**. Observed photosynthesis rates appear to increase in Pol1A^−/−^ mutants.

The decreases in organelle DNA copy number in the mutants raises a question as to whether these changes affect photosynthesis. In previous work with PolIB mutants increases in photosynthesis and related parameters were observed (Cupp and Nielsen, 2013). Current measurements showed an increase in net photosynthesis was observed in 14 dpi PolIA^−/−^ plants (Figure 6). However, we acknowledge that despite careful controls during experimentation, the observed data for Pol1A^−/−^ plants may not be completely accurate. Despite this difficulty in making highly precise measurements, the data suggests that there is an increase in photosynthesis in Pol1A^−/−^ plants, although it cannot be accurately quantified at this time.

### Analysis of PolIA × PolIB partial double mutants

**Figure 7:**
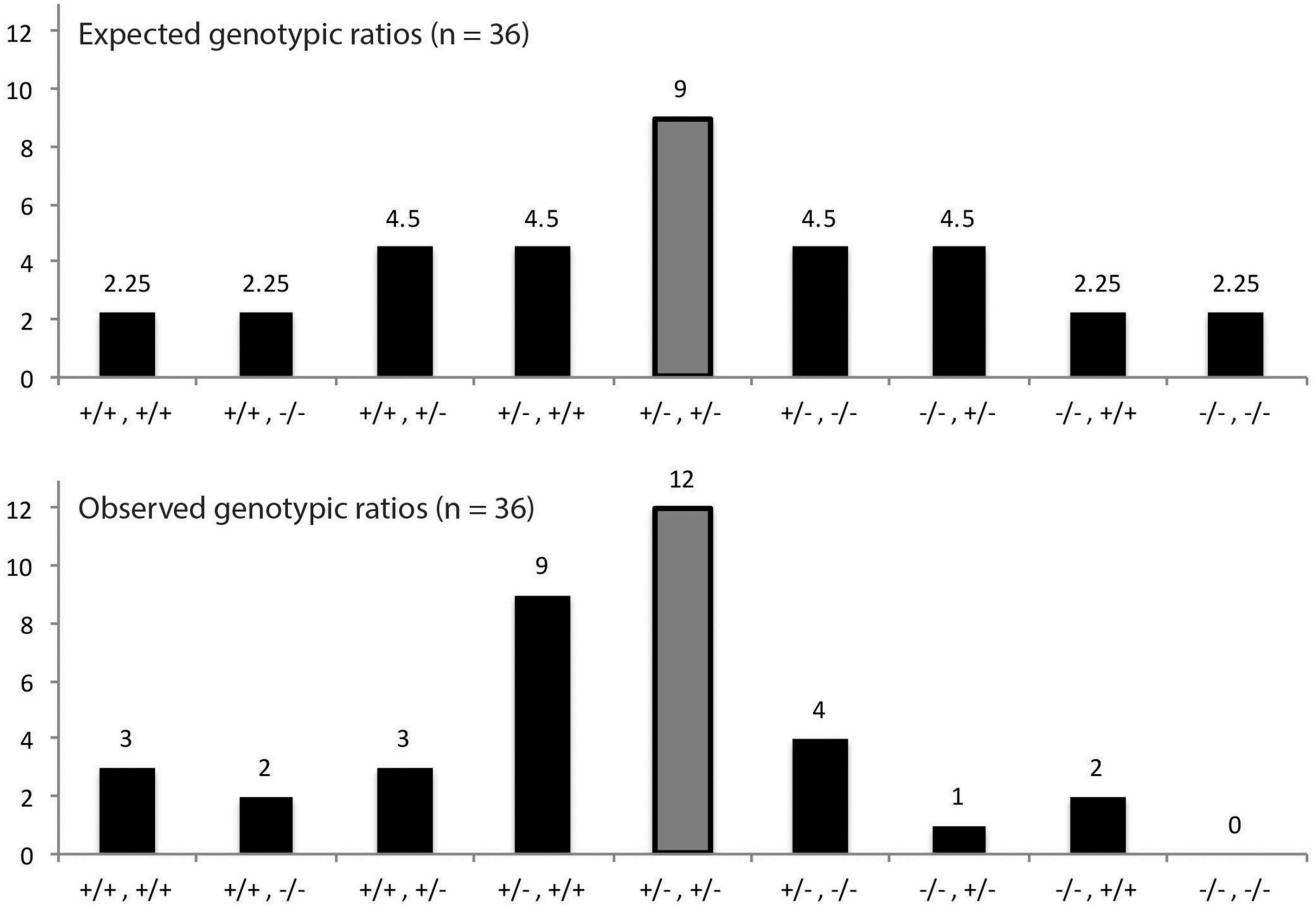
**Proportion of genotypes from DNA PolIA × PolIB crosses**. The results come from 36 plants that were able to successfully grow on soil. The horizontal axes represent the possible genotype combinations starting with PolIA and followed by PolIB (e.g., +/−, +/− represents PolIA^+/−^, PolIB^+/−^, respectively). The middle bar represents the heterozygous combination of genes and is highlighted gray for convenience. Because the results are only from surviving plants, certain genotypes were not observed, such as PolIA^−/−^, PolIB^−/−^ as this combination most likely is lethal to the plant. A particularly interesting genotype was PolIA^+/−^, PolIB^+/+^ which was present in an uncharacteristically high number of plants.

The results of qPCR analysis and previous genotyping experiments led us to believe that certain genotypes would be more beneficial to plant survival than others. To test this theory, we planted seeds on soil in the same manner described above and genotyped all plants that were able to successfully germinate and grow. As expected, none of the surviving plants were homozygous for T-DNA insertions in both DNA polymerase genes as this most likely is lethal to the plant (Figure 7). We also noticed that survival for plants possessing only one functioning DNA polymerase gene was poor. Interestingly we observed strong pressure to maintain both copies of PolIB with at least one functioning copy of PolIA. The pressure to maintain both copies of PolIB suggests higher levels of this polymerase are required to maintain healthy plants.

## Discussion

Analysis of mutations in the genes encoding the organellar DNA polymerases can provide helpful information for understanding their role in chloroplast DNA replication and genome maintenance. However, at the current time analysis of organelle DNA polymerase mutants has apparently only been done for Arabidopsis (Parent et al., 2011; Cupp and Nielsen, 2013) and maize (Udy et al., 2012). In maize it was shown that a single nuclear-encoded chloroplast-localized DNA polymerase (encoded by the *w*2 gene) is responsible for nearly all ctDNA replication (Udy et al., 2012). In contrast, our results show that both PolIA and PolIB are required to maintain normal growth of *A. thaliana* (Figure 2, Supplementary Movie 1, Cupp and Nielsen, 2013).

Both of the previous reports on Arabidopsis focused on PolIB, which indicated effects on mtDNA copy number and mitochondrial structure (Cupp and Nielsen, 2013) and on plastid DNA repair (Parent et al., 2011). In this paper, we have focused on PolIA, and show that it plays a role along with PolIB in ctDNA replication as measured by copy number analysis. This analysis also indicates that PolIA contributes to a lesser extent in mtDNA maintenance. Mutants in each DNA polymerase gene have a limited effect on phenotype, with PolIB plants growing the slowest, while PolIA plants grow only slightly slower than wild type plants.

Analysis of partial double mutants indicates a strong preference for at least one copy of the PolIB gene. As expected, no viable homozygous double mutants were observed, indicating that at least one copy of one of the DNA polymerases is required for growth, although growth is progressively affected by the loss of either the second PolIA or PolIB allele. As mentioned previously, there is a strong pressure to maintain at least two functioning copies of either DNA polymerase gene, and an even stronger pressure to maintain both PolIB genes with at least one functioning PolIA gene. This suggests that PolIB is much more essential to plant survival and may also be needed at higher expression levels to support a healthy plant. This is in line with our previous report that PolIB mutants are haploinsufficient while PolIA is not, which suggests an additive effect of functional PolIB gene copy number (Cupp and Nielsen, 2013).

Expression of the DNA polymerase genes appears to be very high in young developing tissues, especially in meristems (Kimura et al., 2002). PolIA is expressed most abundantly in developing and rosette leaves (Figure 4; Cupp and Nielsen, 2013), which agrees with the data available from online expression databases. In contrast, PolIB is expressed highly (relative to PolIA) in non-photosynthetic tissues (Cupp and Nielsen, 2013). However, both are expressed in all tissues. The higher expression of PolIA in leaves suggests that it may play an important role in ctDNA replication. However, the small effect of a homozygous insertion mutant for this gene on plant growth indicates that the PolIB gene can at least partially complement the PolIA mutation.

A significant increase in PolIA expression was observed in homozygous mutant PolIB plants (Cupp and Nielsen, 2013). In contrast, in homozygous PolIA mutants there is no significant change in PolIB gene expression (Figure 3). PolIA homozygous mutants show an increase in net photosynthesis (Figure 6). Photosynthesis was also affected in PolIB mutants (Cupp and Nielsen, 2013). There may be an inverse relationship between mtDNA levels and net photosynthesis. It may be a decrease in mtDNA, which would affect mitochondrial function, causes a compensatory increase in chloroplast function, including photosynthesis. Thus, while mutants in both genes share some similarities (reduction in growth rate and organelle genome copy numbers and effect on photosynthesis), there are differences in the levels of these effects that strongly suggest different functions for the two DNA polymerases.

**Figure 8:**
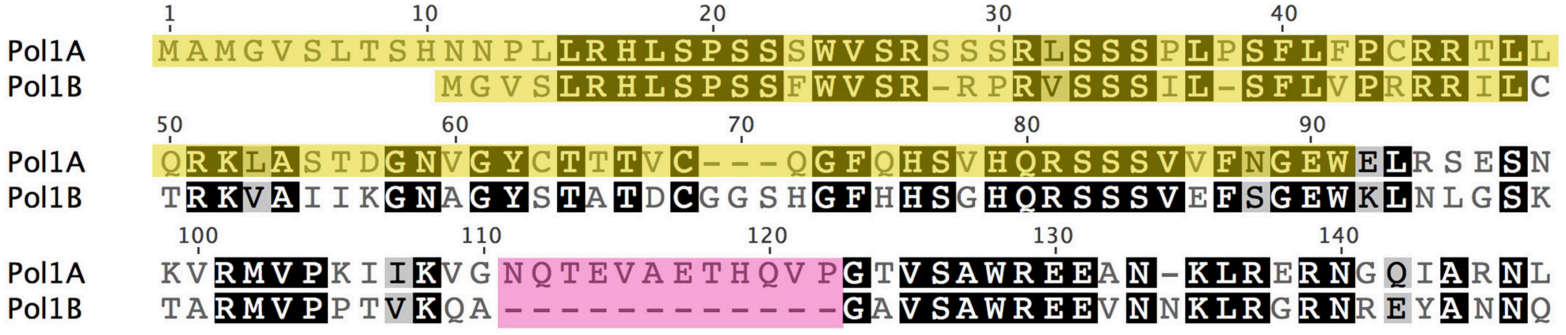
**Predicted signal peptides of PolIA and PolIB and sequence homology in the early region of each polymerase protein**. Predictions of each protein's signal peptide was made using ChloroP. Residues highlighted in yellow represent the predicted signal peptide to be cleaved after localization. Note that despite a much shorter predicted signal peptide, PolIB continues to maintain high homology with PolIA for ~60 more residues. The first region of dissimilarity between the two polymerases is highlighted in pink.

Although, both DNA polymerases have been shown to be dual targeted to chloroplasts and mitochondria, we hypothesize that chloroplasts rely more on Pol1A whereas mitochondria rely more on Pol1B for DNA replication. We hypothesize that a mutation in Pol1B causes increased expression of Pol1A to make up for the loss of function of Pol1B proteins. In the reverse scenario, mutation of Pol1A has a less severe effect, and Pol1B may compensate for loss of function of PolIA without the need for higher PolIB expression. Further supporting this hypothesis are localization predictions based on protein sequence analysis. When the protein sequences for PolIA and PolIB are analyzed by localization prediction programs Target P (Emanuelsson et al., 2007), PCLR, (Schein et al., 2001) and Predotar (https://urgi.versailles.inra.fr/predotar/predotar.html), PolIA is consistently predicted to localize to chloroplasts more strongly than mitochondria while PolIB is most strongly predicted to localize to mitochondria. A summary of these results can be found in Table 1. A more detailed analysis using ChloroP (Emanuelsson et al., 1999) predicts that the first 91 residues of PolIA whereas only the first 36 of PolIB serve as a signal peptide for PolIB, which may help explain the differences in preferred localization. However, PolIB maintains high homology with PolIA beyond its predicted signal for ~60 residues (Figure 8). Thus, while the genes and protein products are highly homologous, they have some significant differences at the N-terminal and other internal regions, contributing to the observation that the two DNA polymerases are not fully redundant to each other.

**Table 1:** **Prediction of PolIA and PolIB organelle localization**.

**Prediction program**	PolIA^*^		PolIB^*^	
	Ct	Mt	Ct	Mt
TargetP	0.928	0.314	0.588	0.741
PCLR	0.995	–	0.915	–
Predotar	0.950	0.100	0.600	0.450

* *Each prediction program returns the likelihood of each resulting protein localizing to either chloroplasts (Ct) or mitochondria (Mt). This prediction is made based on the amino acid sequence of each polymerase*.

In contrast to the computer predictions, both PolIA and PolIB have been shown to be dual-targeted to chloroplasts and mitochondria (Christensen et al., 2005). However, the two DNA polymerases may not be equally localized to both organelles at all stages of plant development. It was reported that plastid localization of PolIA was only obtained when the entire 5′UTR was included in the GFP fusion construct. When the UTR was deleted, initiation of protein synthesis occurred only at the annotated start codon and localization became dual-targeted. The 5′UTR lacks an in-frame upstream start codon, suggesting that an alternate non-AUG start codon was used (Christensen et al., 2005). Localization may vary depending on growth conditions, which could dictate which form of the protein is translated and thus which organelle it is targeted to. This may also play a role in the localization of the proteins when one of the DNA polymerase genes is knocked out in the T-DNA insertion lines. The absence of one DNA polymerase may trigger signal(s) for expression of a form of the other DNA polymerase that can compensate for the mutated enzyme. This could explain some of the slight differences in growth rate and other characteristics between the two mutants. The proposed presence of an alternate mechanism for ctDNA replication could also explain why disruption of one or both of the mapped origins (ori) is not lethal, while some of the linear fragments generated still map near the mapped ori regions (Mühlbauer et al., 2002; Scharff and Koop, 2006). The confirmation and characterization of different replication mechanisms and differential localization of the organellar DNA polymerases during plant development or in response to mutation or stresses deserves further study.

It is interesting that of the four species for which organellar DNA polymerase genes have been characterized, Arabidopsis and tobacco, which are dicotyledonous plants, have two organelle localized DNA polymerases that both appear to be essential for normal growth and replication of chloroplast and mitochondrial genomes. In contrast, maize and rice, which are monocots, appear to have a single DNA polymerase that is responsible for substantially all ctDNA replication. Analysis of organelle DNA polymerases in additional species will be required to determine whether this is a consistent pattern, which would suggest significant differences in the replication machinery for plants from these two lineages.

Chloroplast genome copy numbers per cell are highest in young photosynthetically active leaves. Chloroplast genome copy number varies widely between tissues, ranging from 3 to 275 copies per plastid in leaf cells of different developmental stages (Zoschke et al., 2007; Liere and Borner, 2013). For other species there are 10–400 copies of the chloroplast genome per plastid, translating to 1000–50,000 genome copies per plant cell (also see Boffey and Leech, 1982; Tymms et al., 1983). This number has been given as a compelling basis for chloroplast genetic engineering. Such high copy numbers could theoretically lead to high expression of introduced genes. Indeed, high yields of gene products in engineered chloroplasts have been reported (Grevich and Daniell, 2005; Maliga and Bock, 2011).

## Conclusion

In summary, there are two closely related organelle-localized DNA polymerases in *A. thaliana*. While mutants in either gene have only a slight effect on plant growth and net photosynthesis, the two enzymes do not appear to be fully redundant. Mutation of Pol1B causes a more drastic effect on growth compared to the effect of mutation in Pol1A. This is supported by genome copy number analysis. Mutation of either DNA polymerase causes a similar decrease in ctDNA copy number, while mutation of Pol1B causes a more substantial reduction in mtDNA genome copy number than Pol1A mutation. While knockdown of PolIB resulted in increased expression of PolIA, suggesting compensation for the loss of PolIB (Cupp and Nielsen, 2013), knockdown of PolIA did not lead to any significant change in PolIB expression (this work). However, PolIA mutants exhibit a small increase in net photosynthesis, suggesting some adjustment in plants to the reduction in organelle DNA levels. Analysis of double mutants suggests that while homozygous mutants of either DNA polymerase are still viable, there is a strong pressure to maintain two functioning copies of PolIB or at the least two functioning copies of either DNA polymerase. These findings indicate that both are important for plant organelle genome replication and plant development, and suggest distinct roles for PolIA and PolIB in Arabidopsis. A better understanding of the dynamics and controls of ctDNA copy numbers are important to improve chloroplast genetic engineering to overexpress introduced genes, which is relevant to this special topic issue.

## Funding

This research was supported in part by a Brigham Young University Mentoring Environment Grant and by the National Institutes of Health (USA) (R15GM066787).

## Author contributions

SM and BN designed the experiments and wrote the manuscript. SM performed most of the experiments, analyzed data, prepared figures, and edited the manuscript. BN obtained funding, directed the experiments, performed some of the computational gene analysis, and wrote the manuscript.

### Conflict of interest statement

The authors declare that the research was conducted in the absence of any commercial or financial relationships that could be construed as a potential conflict of interest.

## Supplementary material

Supplementary Movie 1 **Time lapse of WT, Pol1A^**−/−**^, and Pol1B^**−/−**^ plants growing side by side. In this video, plants can be seen growing at slightly different rates based on their mutation**. The plants in the first frame are 7 dpi (days post-imbibition). Black frames represent times when lights were turned off to simulate the night cycle. In total, the plants can be seen growing from 7 dpi to 32 dpi. In this video, no difference is noted between WT and Pol1A^−/−^ plants. However, Pol1B^−/−^ plants can be seen lagging slightly behind WT and Pol1A^−/−^. This delay is most pronounced when plants begin to put up shoots.

Supplementary Table 1 **Primers used for qPCR analysis of mitochondrial and chloroplast genome copy number**.

Supplementary Table 2 **Primers used for RT-PCR analysis of PolIA and PolIB expression**.

Supplementary Table 3 **Primers used for zygosity testing**.
