# Supplementary material for: Chloroplast DNA Copy Number Changes during Plant Development in Organelle DNA Polymerase Mutants
Source: Front Plant Sci. 2016 Feb 4;7:57. doi: 10.3389/fpls.2016.00057 (PMC4740508; doi:10.3389/fpls.2016.00057)
Supplement: Supplementary Table 1 — Primers used for qPCR analysis of mitochondrial and chloroplast genome copy number. [file Table1.PDF]

Primers used for qPCR analysis of mitochondrial and chloroplast genome copy number

Target	Genome	Gene Function
AtRpoTp	Nuclear	DNA-directed RNA polymerase 3
nad9	Mitochondrial	NADH dehydrogenase [ubiquinone] iron-sulfur protein 3
orf25	Mitochondrial	Predicted ATP synthase b subunit
cox1	Mitochondrial	Cytochrome c oxidase subunit 1
psbK	Chloroplast	Photosystem II reaction center protein K
petD	Chloroplast	Cytochrome b6-f complex subunit 4
ndhH	Chloroplast	NAD(P)H-quinone oxireductase subunit H

Primer	Sequence	Tm °C
5’ AtRpoTp	CTGAATGCAGGTCGAAACTCGGG	60.1
3’ AtRpoTp	GCTTGGAAGCCGTCTGCTAGAAC	60.1
5’ nad9	GTGGGAGCGAGAAGTTTGGGATATG	59.4
3’ nad9	GGGTCATCTCAATGGGTTCAGAAACC	59.5
5’ orf25	TCAAAGTGACTCTCGACGGGAGC	60.6
3’ orf25	TGCCACAAATTCGCAAGCTGATCC	60.5
5’ cox1	GAAGTAGGTAGCGGCACTGGG	59.7
3’ cox1	ATTCCAGGTCCACGCATGTTGAAG	59.7
5’ psbK	GTCGCCAAATTGCCAGAGGC	59.7
3’ psbK	CGGCTTGCCAAACAAAGGCTAAGAG	60.7
5’ petD	TATTACGGGGAACCCGCATGG	63.6
3’ petD	GCAAAAGGATCCGCAGGTTCACC	60.9
5’ ndhH	GACTTCCAGGGGGTCCCTATGAG	60.5
3’ ndhH	CCCAACTCCCCTTTTGGAGCTTC	60.2
