# Supplementary material for: Chloroplast DNA Copy Number Changes during Plant Development in Organelle DNA Polymerase Mutants
Source: Front Plant Sci. 2016 Feb 4;7:57. doi: 10.3389/fpls.2016.00057 (PMC4740508; doi:10.3389/fpls.2016.00057)
Supplement: Supplementary Table 2 — Primers used for RT-PCR analysis of PolIA and PolIB expression. [file Table2.PDF]

Primers used for RT-PCR analysis of PolIA and PolIB expression

Primer	Sequence	Tm °C	Target/Purpose
Actin_F	TCCCTCAGCACATTCCTGCAGAT	60.5	Nuclear control/reference
Actin_R	AACGATTCCTGGACCTGCCTC	60.8	for expression comparisons
RTPolA_F	TTCCGGCGTCAAAGTCACGTGC	62.6	PolIA gene
RTPolA_R	TGCACTTCCCTGGACTGGAGTGT	62.4	
RTPolB_F	CCTGAATACCGTTCACGTGCCCA	61.5	PolIB gene
RTPolB_R	AGCCGCACTTCCCTGAACAGGA	63.1	
