# Supplementary material for: Chloroplast DNA Copy Number Changes during Plant Development in Organelle DNA Polymerase Mutants
Source: Front Plant Sci. 2016 Feb 4;7:57. doi: 10.3389/fpls.2016.00057 (PMC4740508; doi:10.3389/fpls.2016.00057)
Supplement: Supplementary Table 3 — Primers used for zygosity testing. [file Table3.PDF]

Primers used for zygosity testing

Primer	Sequence	Tm °C	Target/Purpose
PolA_F	TTGAAGAGCTTCAGCGAGAAG	54.9	PolIA gene
PolA_R	TAGCATGACATGCCTCCTTTC	54.9	
PolB_F	TTACCAAAAGCATCATCCTGG	53.0	PolIB gene
PolB_R	AGAGTTTTCGTGTTCCCCATC	55.0	
Lbb1.3-1	ATTTTGCCGATTTCGGAAC	51.5	T-DNA insert specific primer
